# The Use of Abbreviations in Prescribing

**Published:** 1915-11-20

**Authors:** 


### THE USE OF ABBREVIATIONS IN PRESCRIBING.

A Pharmacopœia has now been published under the name of the "London Insurance Pharmacopœia," and the Panel Committee of the London Insurance Committee have formally adopted it for use in prescribing by practitioners on the panel. The publication of this book will facilitate the use of abbreviations in prescribing, and the Medical Benefit Committee have taken the opportunity to set forth the conditions under which the use of abbreviations is permissible. These instructions were contained in a report submitted to the Insurance Committee at their October meeting. The Commissioners have pointed out that the question is one upon which an arrangement might be made between the Insurance, Panel and Pharmaceutical Committees, subject to approval by the Commissioners, but any arrangement made in this connection would, in effect, amount to a modification of the Committee's agreement with practitioners, although it would not be necessary to obtain the consent of individual practitioners thereto, since it would be open to a practitioner to adhere to the method of prescribing in full or to avail himself of the new method as he thought fit. It is at present the practice of many medical practitioners to order medicines by the use of short or contracted titles indicating formulæ contained in certain standard books of reference, *e.g.* Mist. Gent. et Sodæ B.P.C. In several cases, however, medical practitioners have compiled their own private list of formulæ and supplied chemists in the neighbourhood with copies thereof. Apart from other objections, the latter procedure in effect restricts the right of an insured person to obtain drugs from any chemist on the list, and is one which the Committee cannot recognise. In the opinion of the Medical Benefit Sub-Committee any abbreviations used in prescribing should consist of distinctive names having a definite connotation, rather than numbers or letters, or series of letters, having no apparent meaning except as symbols which would be too liable to lead to mistakes both on the part of practitioners and chemists. The Sub-Committee is therefore of opinion that medical practitioners should not prescribe for insured persons by means of short titles unless such titles indicate formulæ taken from the London Insurance Pharmacopœia or the current editions of standard works of reference, *e.g.* The British Pharmaceutical Codex, Martindale's Extra Pharmacopœia, or the Pharmacopœias of the various London hospitals. Accordingly, at the October meeting the Sub-Committee presented a recommendation to the effect that, as from December 1, 1915, orders for drugs must be written by medical practitioners on the panel so as to secure to insured persons their right to obtain such medicines from any person on the list of persons supplying drugs and appliances; that if such orders are not written in full any abbreviation employed to indicate the formula desired must refer to the current edition of a standard work of reference; that no payment be made by the Committee in respect of prescriptions which cannot be dispensed at sight or upon reference to the London Insurance Pharmacopœia or a standard work by any person supplying drugs and appliances; and that each medical practitioner and chemist on the panel be so informed; and that the concurrence of the Panel and Pharmaceutical Committees and the approval of the Insurance Commissioners be sought thereto. We agree with the policy laid down in this recommendation. While the prescribing of private formulæ may effect a small saving in time the practice is objectionable for at least two reasons, it encourages slipshod methods and prevents the patient from choosing his own chemist.

